# Hypertensive patients’ readiness to use of mobile phones and other information technological modes for improving their compliance to doctors’ advice in Karachi

**DOI:** 10.12669/pjms.311.5469

**Published:** 2015

**Authors:** Mirza Izhar Hussain, Baqir S. Naqvi, Iqbal Ahmed, Nasir Ali

**Affiliations:** 1 Mirza Izhar Hussain, M. Pharm., MBA, Director, Center for Executive Education, Institute of Business Administration, IBA City Campus, Garden/ Kiyani Shaheed Road, Karachi, Pakistan.; 2 Dr. Baqir S. Naqvi, PhD, Professor, Department of Pharmaceutics, Faculty of Pharmacy, University of Karachi, Karachi, Pakistan.; 3 Dr. Iqbal Ahmed, PhD, Professor, Institute of Pharmacy, Baqai University, Karachi, Pakistan.; 4 Nasir Ali, MBA (Supply Chain Management), Research Associate-IBA, Iqra University, Karachi, Pakistan.

**Keywords:** Disease awareness, Patient compliance, Proxy indicators, SMS, Health message

## Abstract

**Objective:**

To determine the use of information technology (IT) & electronic media for improving compliance rate to doctors’ advice in hypertensive patients in Karachi.

**Methods:**

Total 400 persons (200 males & 200 females) were randomly selected in six districts of Karachi. Data was collected through a pretested questionnaire. Following was sample criteria: age above 15 years, living in Karachi and ambulatory. Persons admitted in a hospital, individuals who were doing some physical activity during survey e.g. exercise, labor work etc., individual in stressed condition, non-cooperative individuals – not willing to get BP checked and fill questionnaire, and pregnant women were excluded. Those who did not sign the consent form were also excluded. SPSS was used for data analysis and descriptive statistics was employed for sensitivity analysis.

**Results:**

For healthcare awareness, people look for health programs on radio and TV channels. Short Message Service (SMS) and phone are highly appreciated by patients for reminders. To increase compliance to doctors’ advice, less educated people prefer phone calls over SMS whereas educated individuals favor SMS. Although price of medicine has not emerged as a major contributing factor for non-compliance, discount on medicinal products is highly appreciated by the patients.

**Conclusion:**

The study concludes that there is a widespread awareness of high blood pressure in the sample population of Karachi e.g. 72.5%. People consider reminder message system i.e. Calls and Short Messaging Service (SMS) would help them in improving compliance to doctors’ advice.

## INTRODUCTION

In Pakistan, around 18million people of 15 years and above age are estimated sufferers of high blood pressure. Seventy percent (70%) of them are not aware of this disease. Out of those who are taking medication, only 3% achieve blood pressure control.^1^ Besides hypertension, there is high prevalence of other proxy indicators i.e. high cholesterol, lack of physical activity, central obesity, increased trends in smoking, and high dietary consumption of fat and carbohydrates. Juxtaposing these facts, a large number of cases of stroke, and other cardiovascular events could be projected. Hence disease load of high blood pressure would be significantly high in future. In one study, the factors of non-compliance reported included dose missed due to forgetfulness (56.8%), intentionally dose missed (12.7%), unable to take medicine due to too many tablets (11.6%), poor counseling by doctors (4.6%), and cost issues (3.8%). Other reasons for non-adherence or non-compliance were age, poor awareness and symptomatic treatment.^2^

Cell phone can play its role in improving patient compliance and disease management.^3^ In a clinical trial, hypertension short message service (SMS) was used as tool for the desired blood pressure control. It was reported that its efficacy rate went up to 85% in the trial as compared to 20% in routine practice. It was thought that the success was due to increased communication between patient and physician through SMS.^4^

In the present era, the channels of mass communication have been increased and innovative techniques of knowledge sharing have become more common in the society. Communication channels have become more useful for information sharing and knowledge spreading through different media that focus on the improvement of personal health. Furthermore, efforts towards changing the attitude and behavior, people have become more communicative and effective by the usage and widespread of social marketing methods and techniques. Social marketing is basically the application of marketing practices for the non-profitable social purposes, as Philips Kotler and Gerald Zaltman described it as “the arrangement and implementation of social change by using a potentially strong framework”.^5^

In the public health sector, adoption of the social marketing techniques worldwide has been increasing day by day but there are still many misconceptions and confusion prevalent in the minds of the health professionals. These are basically due to lack of understanding and knowledge about the benefits and influence of social marketing methods.^6^

The recent technological advancements in the field of internet and mobile technology have offered great opportunities for improvement in the public health sector. However, an important aspect of these advancements is still not fully exploited for the benefit of society. Further, these technological developments could also be helpful in improving physician-patient relationship and the follow-up.^7^

The objective of this study was to determine the elements of communication which could improve compliance to physician instructions in a targeted population and help achieve blood pressure control in hypertensive patients. The study also aimed to find effectiveness of current communication modes for improving patient’s compliance.

## METHODS

The population of Karachi is approximately 18 Million and is increasing continually and thought it has surpassed 20 million but still the official figures show around 14.5 million.^8^,^9^ Estimated population of hypertensive patients is about one million in the city. The study employed Stratified Random Sampling technique for a sample size of 400 residents comprising 200 males and 200 females for the June to August 2012 study period.

For the purpose of sample selection, the study established inclusion and exclusion criteria. Inclusion criterion included (1) above 15 years of age, (2) Karachi resident; and (3) ambulatory patients. Whereas, exclusion criteria excluded (1) persons admitted in healthcare facilities e.g. medical centers or hospitals, (2) individuals who were doing some physical activity during the survey e.g. exercise, labor work etc., which can change their blood pressure,(3) individual observed to be in stressed condition, (4) who are not willing to cooperate for blood pressure check and fill questionnaire, (5) who do not want to sign survey consent form; and (6) pregnant women.

For the purpose of data collection, a survey was performed using a questionnaire developed in light of the literature review ^10^^-^^13^ Statistical Package for Social Sciences (SPSS) was used for data analysis and descriptive statistics used for analyzing the data regarding sensitivity analysis, frequency of hypertensive, and normotensive and awareness level between the hypertensive and normotensive.

## RESULTS

Descriptive statistics was summarized in terms of frequencies and percentages for qualitative/categorical variables (gender, occupation, education, income grouping, marital status, and smoking habits). Table-I shows demographics of the sample age, occupation, education, monthly income, marital status, and smoking habit.

Table-II shows the descriptive statistics about the awareness of hypertension among male and female normotensive and hypertensive respondents. The result shows that among 308 normotensive respondents, 209 (67.9%) were aware of hypertension which included 92 (44%) male and 117 (56%) female while the remaining normotensive respondents were unaware of their hypertension i.e. 99 (32.1%) that included 63 (63.6%) male and 36 (36.4%) female.

## DISCUSSION

Total sample size comprised 400 of which 200 were males and 200 females. The sensitivity analysis of the data (Table III & IV) shows the uncertainty of the responses between their understanding and the actual situation of their disease awareness.

About 83 (20.8%) of the selected individuals self-reported to be hypertensive of which 43 were (51.8%) males and 40 (48.2%) were females, which means hypertension was prevalent almost equally in both the genders. The sensitivity analysis reveals that either control is established in 9 (2.9%) including 7 (77.8%) males and 2 (22.2%) females, or those people who thought they had hypertension but did not have. Of those reporting to be non-hypertensive, 12 (14%) were found hypertensive which meant they were not aware of their hypertensive silent killer condition or thought they were healthy but were at risk of hypertension. These (14%) included 5 (41.7%) male and 7 (58.3%) female.

**Table-I:** Patients’ characteristics according to gender

	*Overall(n=400)*		*Male(n=200)*		*Female(n=200)*	
	*No.*	*%*	*No.*	*%*	*No.*	*%*
Age						
15 – 24	51	12.8	25	12.5	26	13.1
25 – 34	106	26.6	56	28.0	50	25.1
35 – 44	122	30.6	58	29.0	64	32.2
45 – 54	73	18.3	36	18.0	37	18.6
55 – 64	36	9.0	18	9.0	18	9.0
65 – 74	11	2.8	7	3.5	4	2.0
Occupation						
Business	101	25.3	89	44.5	12	6.0
Private Job	78	19.5	53	26.5	25	12.5
Govt. Job	9	2.3	8	4.0	1	0.5
House wife	137	34.3	0	0.0	137	68.5
Labor	13	3.3	13	6.5	0	0.0
Student	42	10.5	17	8.5	25	12.5
Retired	20	5.0	20	10.0	0	0.0
Education						
None	33	8.3	15	7.5	18	9.0
Primary to middle	18	4.5	4	2.0	14	7.0
Matric	56	14.0	32	16.0	24	12.0
Intermediate	82	20.5	36	18.0	46	23.0
Graduate	177	44.3	96	48.0	81	40.5
Masters	34	8.5	17	8.5	17	8.5
Monthly Income (Rs/=)						
Up to 10,000	43	10.8	17	8.5	26	13.0
10001 - 20,000	99	24.8	49	24.5	50	25.0
20,001 - 30,000	87	21.8	47	23.5	40	20.0
30,001-40,000	98	24.5	48	24.0	50	25.0
>50,000	73	18.3	39	19.5	34	17.0
Marital status						
Married	310	77.5	153	76.5	157	78.5
Single	90	22.5	47	23.5	43	21.5
Smoker						
Yes	46	11.5	43	21.5	3	1.5
No	354	88.5	157	78.5	197	98.5

**Table-II:** Awareness about hypertension according to gender

		***Awareness about Hypertension***		
		***Normotensive***	***Hypertensive***	***Total***
Male	Yes	92	36	128
	%	59.4	80	64
	No	63	9	72
	%	40.6	20	36
	Total	155	45	200
Female	Yes	117	45	162
	%	76.5	95.7	81
	No	36	2	38
	%	23.5	4.3	19
	Total	153	47	200
Overall	Yes	209	81	290
	%	67.9	88	72.5
	No	99	11	110
	%	32.1	12	27.5
	Total	308	92	400

**Table-III:** Sensitivity Analysis

		***Sensitivity Analysis***		
		***Yes***	***No***	***Total***
Male	Yes	36	7	43
	%	87.8	4.4	21.5
	No	5	152	157
	%	12.2	95.6	78.5
	Total	41	159	200
Female	Yes	38	2	40
	%	84.4	1.3	20.0
	No	7	153	160
	%	15.6	98.7	80.0
	Total	45	155	200
Overall	Yes	74	9	83
	%	86.0	2.9	20.8
	No	12	305	317
	%	14.0	97.1	79.3
	Total	86	314	400

**Table-IV:** Results for Hypertension Awareness and Prevalence Survey

***Acknowledgement of Hypertension by the Individual***	***Awareness & Prevalence Status of Hypertension***		***Total***
	***With*** ***High Blood Pressure***	***Without*** ***High Blood Pressure***	
Know	74	9	83
Don’t know	12	305	317
Total	86	314	400

Sample (N) = 400.

Proportion of the population with awareness of hypertension (p)= 0.1850.

Sensitivity = 0.8605, Specificity = 0.9713,

Positive Predictive Value (PPV) = 0.8916, Negative Predictive Value (NPV) = 0.9621

Likelihood of Positive Ratio =30.0207, Likelihood of Negative Ratio = 0.1437

**Table-V:** Modes of communication to improve compliance to physician instructions among hypertensive patients

		***Hypertension***		***Total*** ***(%)***
		***Not Controlled***	***Controlled***	
Do you receive any other alerts (messages) regarding (intake of) your medicine?	No	61	20	81 (98.8%)
	Yes (call for checkup)	1	0	1 (1.2%)
Which sort (mode) of communication or alert you think will help you most to take your medicine?	Short Messaging Service (SMS)	5	2	7 (8.5%)
	Calls	42	11	53 (64.6%)
	SMS & call	12	4	16 (19.5%)
	SMS, call and email	1	1	2 (2.4%)
	Emails	0	1	1 (1.2%)
	Other please specify	1	1	2 (2.4%)
	Face-to-Face television program	1	0	1 (1.2%)

**Fig.1:**
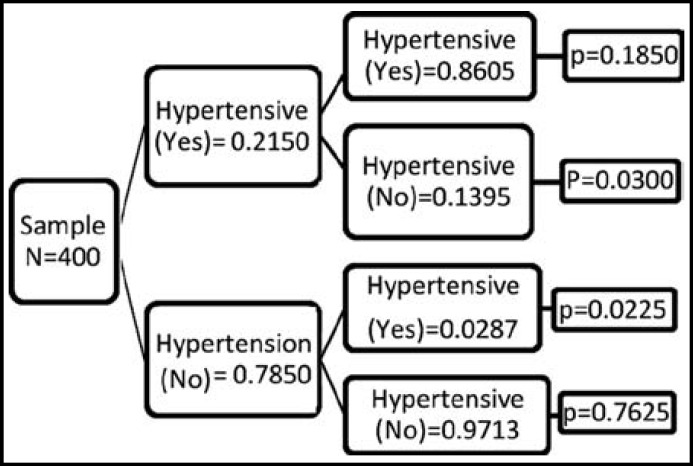
Probability Tree: Patients randomly screened for awareness & prevalence of high blood pressure.

The probability tree of survey for determining awareness and prevalence of hypertension applied to the population is presented in Fig.1.The first branch indicates the proportion of population with awareness and knowledge of their disease e.g. hypertension; and the second branch reflects the sensitivity and specificity characteristics of this work. The results indicate that a person, at random, from this population has:

- A probability of 0.1850 of being afflicted with hypertension and acknowledging positive (a true positive result)
- A probability of 0.0300 of being afflicted with hypertension and acknowledging negative (a false negative result)
- A probability of 0.0225 of not being afflicted with hypertension and acknowledging positive (a false positive result)
- A probability of 0.7625 of not being afflicted with hypertension and acknowledging negative (a true negative result)

The Table-IV presents degree of accuracy of awareness about having hypertension on overall basis (he data is derived from Fig.1). The total awareness of having hypertension is 0.1850. Further, the sensitivity of this survey is equal to the proportion of subjects who have disease and know about it, which in this case is 0.8605. Specificity of the proportion of the subjects who do not have the disease and who know they do have disease (accurate knowledge), is 0.9713.

The positive predictive value (PPV) is 0.8916, whereas the negative predictive value (NPV) is 0.9621. Thus it might be expected that only about 89% of subjects who say they have high blood pressure are actually hypertensive. However, a subject who denies suffering from high blood pressure may be hypertension free (at least 96% of the time) in this population.

The data in Table V shows that not a single hypertensive subject in the population receives any message for taking the medicine for achieving higher control rate of hypertension. When inquired how would they prefer to receives one sort of alerts, their response was: Phone calls on their cellular phones (64.6%); SMS (8.5%); and e-mail (1.4%) for improving compliance to doctor’s advice and reminding medicine intake. Nineteen–and-a-half percent (19.5%) preferred both SMS and mobile phone calls, whereas only 2.4% desired by calls, SMS, and e-mails. Phone-Calls are preferred because:

a) Mobile Phones are common and available to almost everyone, whereas internet is not accessible to every person.

b) Phone calls are more convenient than e-mails.

c) Elderly people have difficulty in reading SMS and accessing their e-mails.

d) Less educated people also prefer phone calls as they have difficulty in following the messages sent through SMS.

e) The subjects look for a chance to discuss their concerns.

f) They gain more confidence through two-way communications.

g) Personal touch of phone call that has greater impact than that of SMS or e-mail.

Significantly high number of subjects asked for increasing air time of health and disease related programs on television and radio for improving disease awareness of general public. For improving adherence rate to doctors’ advice and thus control of blood pressure, discount on medicine and regular B.P. monitoring could be used. Discount on medicine was more emphasized by the patients as it would help in improving compliance. Other suggestions included increased discussion session and social support to the patients. By social support they meant establishment of hypertensive care centers, call centers for answering queries and educating patients, and continuous public education lectures on hypertension.

## CONCLUSION

The study concludes that there is a widespread awareness of hypertension not only among hypertensive patients but also among normotensive as well, in both genders in Karachi. However, rate of control of hypertension is just 24.4%; 98.8% do not receive any reminder for improving drug intake, where as 1.2% do receive reminder message for medical checkup. For improving disease awareness, most of the subjects prefer health awareness programs on television and radio. 87.5% of subjects prefer phone calls alone (64.6%), with SMS (19.5%), and with SMS & e-mail (2.4%). SMS is preferred by 8.5% subjects only. This shows that phone calls could be beneficial for better rate of control of high blood pressure. It is recommended that a structured study should be designed to find out how this mode could be used effectively to improve patients’ adherence to doctors’ advice.

## Authors’ Contribution:

**MIH: **conceived, designed, prepared manuscript, statistical analysis of data, and statistical inference. 

**BSN, MIH & NA**: Collected data.

**NA: **Referencing and arranged data.

**IA:** Design modification.

**BSN**: Reviewed and final approval of manuscript.

**MIH**: Takes responsibility and accountable for all aspects of the work in ensuring.
